# An assessment of burden of hospital-acquired pneumonia among abdominal surgical patients in tertiary university hospital in Serbia: A matched nested case-control study

**DOI:** 10.3389/fmed.2022.1040654

**Published:** 2022-12-09

**Authors:** Ðorde Taušan, Nemanja Rančić, Zoran Kostić, Nenad Ljubenović, Bojan Rakonjac, Vesna Šuljagić

**Affiliations:** ^1^Pulmonology Clinic, Military Medical Academy, Belgrade, Serbia; ^2^Center for Clinical Pharmacology, Military Medical Academy, Belgrade, Serbia; ^3^Medical Faculty, University of Defence, Belgrade, Serbia; ^4^Clinic for General Surgery, Military Medical Academy, Belgrade, Serbia; ^5^Institute of Epidemiology, Military Medical Academy, Belgrade, Serbia; ^6^Institute of Medical Microbiology, Military Medical Academy, Belgrade, Serbia; ^7^Department of Healthcare-Associated Infection Prevention and Control, Military Medical Academy, Belgrade, Serbia

**Keywords:** antibiotics, drug consumptions, abdominal surgery, risk factors, in-hospital mortality, *Acinetobacter* spp.

## Abstract

**Background:**

In the population of abdominal surgical patients hospital-acquired pneumonia (HAP) significantly increases morbidity and mortality.

**Patients and methods:**

Through regular hospital surveillance of patients who received abdominal operations, we identified postoperative HAP from 2007 to 2019. In an initial nested case-control study, every surgical patient with HAP was compared with three control patients without HAP. Control patients were matched to the cases by age, gender, the American Society of Anesthesiologists score, and type of surgical operation. Also, the patients with HAP, who died were compared with those who survived.

**Results:**

Multivariate logistic regression analysis (MLRA) revealed that other postoperative infections, length of intensive care unit stay, use of H2RA, use of PPI/ H2RA, multiple transfusion, and use of vancomycin in surgical prophylaxis were independent RFs for occurrence of HAP. Also, MLRA identified that age, lenght of hospital stay, use of mechanical ventilation and ceftriaxone in HAP therapy were indepedenttly associated with poor outcome of HAP. All *Acinetobacter baumannii* isolates were resistant to aminoglycoside antimicrobial agents and showed carbapenem resistance. The most frequently used antibiotics in patients with HAP and without HAP were vancomycin and metronidazole, respectively.

**Conclusion:**

Our study provided an insight into the burden of HAP in abdominal surgical patients, and highlighted several priority areas and targets for quality improvement.

## Introduction

Hospital-acquired pneumonia (HAP) is a challenging problem associated with healthcare in hospitals worldwide (1, 2). The reported rate of HAP varied according to the type of population studied, type of hospital ward, and length of hospital stay (LOS) (2–5). Ventilator-associated pneumonia (VAP) refers to HAP that develops among patients on ventilators (6). Both HAP and VAP are frequent complications of hospital care, accounting for almost 22% of all hospital-acquired infections (HAI) in US and European point-prevalence surveys (7, 8). However, a significant difference in the density of incidence of VAP was observed between US and EU intensive care units. The European Centre for Disease Prevention and Control (ECDC) reported that the average rate of VAP in the EU was 8.9 episodes/1,000 days of MV (9) substantially higher than in the USA, 1-2.5 cases/1000 days according to the National Healthcare Safety Network (NHSN) (2013) (10). HAP is the second most common HAI, after urinary tract infection, but the most dangerous with the highest mortality (11, 12). Recently, mortality of 9.2% was reported in HAP acquired in a long-term care, subacute, or intermediate health care facility following recent hospitalization; or after chronic dialysis (12). Previously conducted studies indicate that risk factors (RFs) for HAP include mechanical ventilation (MV) for >48 h, residence in an intensive care unit (ICU), duration of ICU or hospital stay, the severity of underlying illness, and presence of comorbidities (13). HAP is a common complication that can adversely affect the outcomes after surgery. In the population of abdominal surgical patients, HAP significantly increases morbidity and mortality, prolonging the duration of hospitalization and increasing the costs of treatment (14–17). Studies using different methodologies conducted in different parts of the world during the first two decades of this century indicate a different rate of HAP in patients undergoing intra-abdominal surgery. Thompson et al. studied 618,495 patients who underwent an intra-abdominal operation from the National Inpatient Sample database in the USA, over a 1-year period (January 2000 to December 2000) and reported that the incidence of HAP was greatest in patients undergoing a gastrostomy (109 per 1,000 procedures), followed by gastrectomy (80.2 per 1,000 procedures), small bowel resection (24.6 per 1,000 procedures), colorectal resection (21 per 1,000 procedures), colostomy (17.9 per 1,000 procedures), exploratory laparotomy (16 per 1,000 procedures), renal transplant (12.1 per 1,000 procedures), ileostomy (11,3 per 1,000 procedures cases), and cholecystectomy (7.4 per 1,000 procedures) (15). Prospective surveillance of all elective gastric resections by surgeons in ten affiliated hospitals in Japan, from May 2001 to May 2005, determined that 3.6% of operative procedures were complicated by HAP (14). Kochi et al. retrospectively investigated 1,473 patients ≥80 years of age who underwent surgery for colorectal cancer between 2003 and 2007 and found HAP with frequency of 24.1 per 1,000 open procedures and 6.0 per 1,000 laparoscopic procedures (18). During the period 2016-2019, Baba at al. investigated the clinical features of patients with HAP after general and digestive surgery and found that esophagectomy accounted for 33% of patients with registred HAP, however in the total number of operated patients, this type of operation represented only 5 % (19). Recently published matched 1:1 case-control study, conducted on adult patients who underwent surgery between January 2020 and June 2020, showed that 2.69% of 1,300 patients after general and digestive surgery acquired HAP (20). Our earlier study showed that 1.4% of all abdominal surgery patients developed HAP in the postoperative period (17). In the present study, we aimed to determine the burden of HAP (cumulative incidence rate, all-cause hospital mortality rate of patients with HAP, risk factors for occurrence and outcome of HAP, antibiotic consumption, microbiological causes, and antimicrobial resistance) in patients with abdominal surgical procedures hospitalized in a tertiary hospital in Belgrade (Serbia).

## Methods

The Military Medical Academy (MMA), Belgrade, Serbia, a teaching hospital of the University of Defence, is a 1000-bed tertiary healthcare center with 27 departments according to medical specialities. The Clinic for General Surgery is a 72-bed department of the MMA. Through regular hospital surveillance of patients who received abdominal operations, we identified postoperative HAP and other HAIs during the study period, from 1st January 2007 to 31st December 2019. Patient data were obtained by patient-based active surveillance methods. Follow-up, registration, and examination of HAP/HAI were carried out by the Department of Healthcare-associated Infection Prevention and Control. For the cohort of abdominal surgical patients, we collected data on the following variables: patients characteristics existing before operative procedures—age, gender, the presence of underlying diabetes mellitus, the American Society of Anaesthesiologists (ASA) score, preoperative infection, other postoperative infection, malignancy, factors related to health care including the type of operative procedure, the LOS, ICU admission, the length of ICU stay, central vascular catheter (CVC), MV, histamine-2-receptor antagonists (H2RAs) use, proton-pump inhibitors (PPIs) use, both H2RAs and PPIs use, red blood cell transfusion, outcome of treatment (survive/death) and characteristics of operative procedure—elective surgery, upper abdominal surgery, duration of operation in minutes, class of contamination of the surgical site, drainage, duration of drainage in days, surgical site infection (SSI). We analyzed prophylactic and therapeutic use of antibiotics (cefazolin, cefuroxime, ceftriaxone, cefepime, amikacin, ciprofloxacin, levofloxacin, ertapenem, imipenem, meropenem, linezolid, metronidazole, amoxicillin, piperacillin, teicoplanin, vancomycin, trimethoprim). Also, we analyzed use of colistin, tigecycline only for therapeutic purpose. Data were analyzed in two different ways. In an initial nested case-control study (in a cohort study of abdominal surgical patients), every surgical patient with HAP was compared with three control patients without HAP. Control patients were matched to the cases by age (±5 years), gender, ASA score, and type of surgical operation. A second analysis was done on the group of patients with HAP, those who died were compared with those who survived. Patients with pneumonia that developed after postoperative respiratory failure and preoperative pneumonia and were excluded.

All samples (broncho-alveolar lavage, quantitative culture of low respiratory tract specimen, e.g., endotracheal aspirate, with a threshold of 10^6^ CFU/ml, positive blood culture not related to another source of infection, non-quantitative culture of low respiratory tract specimen, e.g., endotracheal aspirate and sputum) taken from patients with HAP were processed at the Institute of Microbiology MMA, according to standard procedures for different samples. After cultivation the identification of isolated strains was confirmed by tests of two different manufacturers: BBL Crystal ID Kit (Becton Dickinson, Sparks Glencoe, MD, USA), and VITEK 2 ID (bioMérieux, Marcy l'Etoile, France). Interpretation of antimicrobial resistance testing is in accordance with the European Committee on Antimicrobial Susceptibility Testing (EUCAST) recommendations. For each isolate shown, the current versions of EUCAST were valid in the year when resistance was determined. At the time of testing isolates of *Acinetobacter baumanii*, the minimum inhibitory concentration was determined by gradient test strips as recommended by EUCAST.

### Definitions

All patients were assessed before the operation by anaesthesiologists for the ASA score (21). The National Research Council operative site classification was used by the surgeon to class surgical wounds as clean, clean/contaminated, contaminated, and dirty/infected (22). Surgical Site Infection (SSI) is defined according to the Centre for Disease Control and Prevention/National Healthcare Safety Network (CDC/NHSN) surveillance definitions (23). Multiple transfusions are defined as more than one pack of red blood cells. Post-discharge surveillance was not performed.

Patients with HAP were treated in consultation with a competent pulmonologist and infectious diseases specialist.

The diagnosis of pneumonia was made in the patient with “new lung infiltrates plus clinical evidence that the infiltrate is of an infectious origin, which includes the new onset of fever, purulent sputum, leukocytosis, and decline in oxygenation” (24). HAP was defined as pneumonia that occurs 48 h or more after hospital admission and not incubating at the admission time and was diagnosed by the consultative specialist of pulmonology based on the presence of radiographic shadowing or scanner findings (1, 6, 23, 25). In this study HAP was defined as pneumonia that occurs 48 hours and more after intraabdominal surgical procedure, also. HAP was stratified into five disease codes, from PN1 to PN5, corresponding to different degrees of microbiological evidence according to European Centre for Disease Prevention and Control (ECDC) (26).

### Statistical analysis

Data analyses were performed with IBM SPSS Statistics for Windows, Version 26.0 (IBM Corp. Armonk, NY, USA; 2019). Results were expressed as the mean ± standard deviation (X±SD), median with interquartile range (M-IQR) or a proportion of the total number of patients. The Chi-square-test or Fischer's exact test was used for categorical variables and relative risk, and their corresponding 95% confidence intervals (CI) were calculated. For parametric continuous variables, mean values were compared using an independent t-test. For nonparametric continuous variables, the Mann-Whitney U test was used. RFs independently associated with HAP were identified by the stepwise logistic regression analysis of variables selected by univariate analysis, with a limit for entering and removing variables at 0.05.

Cumulative incidence rate (CIR) was defined as the number of HAP per 1,000 specific abdominal operative procedures. The all-cause hospital mortality rate was defined as the number of deaths per 100 patients with HAP.

Antibiotic consumption was presented according to standard the World Health Organization (WHO) Anatomical Therapeutic Chemical/Defined Daily Dose (ATC/DDD) methodology for the in-bed-patients. In this calculation method, the form used for in-bed patients is the ratio of the total DDD per 100-bed-days. Results were obtained for the following groups of antibiotics, according to the ATC codes: J01DB04 cefazolin, J01DC02 cefuroxime, J01DD04 ceftriaxone, J01DE01 cefepime, J01GB06 amikacin, J01MA02 ciprofloxacin, J01DH03 ertapenem, J01DH51 imipenem and cilastatin, J01MA12 levofloxacin, J01XX08 linezolid, J01DH02 meropenem, J01XD01 metronidazole, J01CA04 amoxicillin, J01CA12 piperacillin, J01XA02 teicoplanin, J01EA01 trimethoprim, J01XA01 vancomycin, J04AB02 rifampicin, J01MA14 moxifloxacin, J02AC01 fluconazole, J01XB01 colistin, J01FA01 erythromycin and J01AA12 tigecycline.

The informed written consent was obtained from all participants. The Ethics Committee of the Military Medical Academy approved the research protocol (MF VMA 14032018).

## Results

### Cumulative incidence

Overall 154 or 1.6% of abdominal surgical patients developed HAP in the postoperative period. In the sample of 9731 abdominal operations, colorectal surgery was the most common operative procedure, accounting for 4224 or 43.4 % (Table 1). The frequency of specific abdominal surgical procedures and HAP CIR in the study population are shown in Table 1. The CIR of HAP was greatest in patients undergoing small bowel surgery (40.3 per 1,000 surgical procedures), followed by exploratory laparotomy (33.0 per 1,000 surgical procedures), gastric surgery (29.3 per 1,000 surgical procedures), bile duct or liver surgery (23.8 per 1,000 surgical procedures), pancreatic surgery (16.2 per 1,000 surgical procedures), spleen surgery (15.5 per 1,000 surgical procedures), colorectal surgery (9.0 per 1,000 surgical procedures), gallbladder surgery (5.1 per 1,000 surgical procedures), and appendix surgery (1.0 per 1,000 surgical procedures).

**Table 1:** Number and percentage of specific intra-abdominal surgical procedures among all intra-abdominal surgical procedures and hospital-acquired pneumonia (HAP) rate in the study population.

**Surgical procedure**	**Frequency**	**% sample**	**Number of HAP**	**CIR of HAP per 1,000 surgical procedures**
Colorectal surgery	4,224	43.4%	38	9.0
Appendix surgery	986	10.1%	1	1.0
Small bowel surgery	1,041	10.7%	42	40.3
Gastric surgery	989	10.2%	29	29.3
Gallbladder surgery	865	8.9%	5	5.1
Bile duct and liver surgery	671	6.9%	16	23.8
Pancreatic surgery	371	3.8%	6	16.2
Exploratory Laparotomy	455	4.7%	15	33.0
Spleen surgery	129	1.30%	2	15.5
Total	9,731	100%	154	/

HAP, hospital-acquired pneumonia; CIR, cumulative incidence rate.

One hundred and forty surgical patients with HAP were enrolled in the nested case-control study. A random sample of 420 control patients matched by age (±5 years), gender, ASA score, and type of surgical operation was selected from a total of 9,577 potentially matched controlled subjects. Due to the inability to select appropriate controls for the remaining fourteen patients with HAP, they were excluded from further study.

### Risk factors for the acquisition of HAP

The patients with HAP had a mean age of 64.46 ± 14.88 and 59.3% were male. Patients' characteristics, procedures during hospitalization, and characteristics related to the surgical procedure performed in the patients with pneumonia and patients without pneumonia according to univariate logistic regression analysis (ULRA) are shown in Table 2. According to ULRA, the following variables had significant differences between the groups of patients with or without pneumonia: preoperative infections, other postoperative infections, malignancy, treatment outcome, LOS, hospitalization in ICU, length of ICU stay, CVC, MV, use of H2RA, use H2RA/PPI, multiple transfusion, elective surgery, class of contamination, duration of drainage, SSI, use of ceftriaxone, use of imipenem, use of meropenem, use of piperacillin and use of vancomycin. Multivariate logistic regression analysis (MLRA) revealed that other postoperative infections (*p* <0.001; OR: 51.90; 95%CI: 10.92–246.59), length of stay in ICU (*p* = 0.002; OR: 1.17; 95% CI: 1.06–1.29), use of H2RA (*p* = 0.007; OR:4.17; 95%CI: 1.47–11.84), use of PPI/ H2RA (*p* = 0.049; OR:6.53; 95%CI: 1.01–42.33), multiple transfusion (*p* = 0.009; OR: 3.76; 95%CI: 1.39–10.16) and use of vancomycin (*p* = 0.011; OR: 5.69; 95%CI: 1.48–21.78) were independent RFs for HAP.

**Table 2:** Potential risk factors for acquisition of hospital-acquired pneumonia (HAP) in abdominal surgical patients: results of univariate logistic regression analysis and multivariate regression analysis.

**Variables**	**With HAP (*N* = 140)**	**Without HAP (*N =* 420)**	**ULRA Crude RR (95%CI)**	**P**	**MLRA Adjusted OR**	**P**
**Patient characteristics**						
Age (X±SD)	64.46 ± 14.88	64.81 ± 14.22	1.00 (0.98–1.01)	0.803		
Male (number, %)	83 (59.3)	249 (59.3)	0.99 (0.67–1.46)	0.952		
Diabetes mellitus (number, %)	5 (3.6)	15 (3.6)	0.99 (0.35-2.78)	0.989		
ASA (number, %)			1.02 (0.63–1.64)	0.875		
2	29 (20.7)	89 (21.2)				
3	99 (70.7)	298 (71.0)				
4	12 (8.6)	33 (7.9)				
Preoperative infection (number, %)	10 (7.1)	11 (2.6)	0.35 (0.15–0.88)	0.020	8.50 (0.38–187.96)	0.175
Other postoperative infection (number, %)	92 (65.7)	10 (2.4)	0.01 (0.01–0.03)	<0.001	51.90 (10.92–246.59)	<0.001
Malignancy (number, %)	24 (27.9)	117 (45.3)	2.14 (1.26–3.65)	0.005	1.42 (0.56–3.61)	0.462
Treatment outcome (number, %)	73 (52.1)	48 (11.4)	0.12 (0.08–0.19)	<0.001	0.80 (0.29–2.30)	0.680
**Procedures during hospitalization**						
Length of hospital stay (in days) (M-IQR)	38.00 (23.50–56.00)	19.00 (12.00-29.00)	1.05 (1.04–1.06)	<0.001	1.01 (0.99–1.03)	0.533
Hospitalization in ICU (number, %)	124 (88.6)	156 (37.1)	13.22 (7.58–23.07)	<0.001	0.63 (0.22–1.82)	0.397
Length of ICU stay (in days) (M-IQR)	15.00 (3.00–28.75)	0.00 (0.00–1.00)	1.21 (1.16–1.25)	<0.001	1.17 (1.06–1.29)	0.002
Central Venous Catheter (number, %)	121 (86.4)	251 (59.8)	4.32 (2.57-7.28)	<0.001	0.71 (0.27–1.91)	0.502
Mechanical ventilation (number, %)	92 (65.7)	29 (6.9)	26.12 (15.63–43.65)	<0.001	1.37 (0.35–5.37)	0.646
H2RA (number, %)	75 (53.6)	153 (36.4)	2.04 (1.39-3.00)	<0.001	4.17 (1.47–11.84)	0.007
PPI (number, %)	74 (52.9)	183 (43.6)	1.43 (0.98–2.10)	0.067		
Acid suppressive medications (H2RA or PPI)(number, %)	131 (93.6)	332 (79.0)	3.89 (1.90–7.95)	<0.001	6.53 (1.01–42.33)	0.049
Multiple transfusion (number, %)	127 (90.7)	156 (37.1)	16.66 (9.11–30.48)	<0.001	3.76 (1.39–10.16)	0.009
**Characteristics depends of surgery procedure**						
Elective surgery	87 (62.1)	297 (74.3)	1.79 (1.19-2–68)	0.005	1.72 (0.65–4.54)	0.273
Upper abdominal surgery (number, %)	55 (39.3)	163 (38.8)	1.04 (0.70–1.53)	0.849		
Duration of operation (in minutes) (M-IQR)	117.50 (81.25–167.50)	110.00 (80.00–155.00)	1.00 (1.00–1.00)	0.870		
Class of contamination (number, %)			0.46 (0.01–0.34)	<0.001	1.79 (0.11–30.10)	0.432
Clean	1 (0.7)	28 (6.7)				
Clean/contaminated	44 (31.4)	265 (63.1)				
Contaminated	11 (7.9)	18 (4.3)				
Dirty/infected	84 (60.0)	109 (26.0)				
Drainage (number, %)	135 (96.4)	389 (92.6)	2.17 (0.83–5.69)	0.116		
Drainage (in days) (M-IQR)	12.00 (7.00–18.00)	7.00 (5.00–10.00)	1.10 (1.07–1.14)	<0.001	1.03 (0.98–1.09)	0.233
Surgical site infection	31 (22.1)	30 (7.1)	3.82 (2.22–6.56)	<0.001	1.93 (0.61–6.07)	0.260
**Antibiotics (surgical prophylaxis)**						
Cefazoline	3 (2.1)	27 (6.4)	0.32 (0.09–1.06)	0.062		
Cefuroxime	46 (32.9)	160 (38.1)	0.78 (0.52–1.17)	0.172		
Ceftriaxone	33 (23.6)	183 (43.6)	0.40 (0.26–0.61)	<0.001	0.49 (0.18–1.36)	0.169
Cefepime	4 (2.9)	0	/	0.999		
Amikacin	18 (12.9)	72 (17.1)	0.71 (0.41–1.23)	0.222		
Ciprofloxacin	5 (3.6)	17 (4.0)	0.87 (0.32–2.41)	0.791		
Levofloxacin	6 (4.3)	0	/	0.999		
Ertapenem	15 (10.7)	25 (6.0)	1.88 (0.96–3.68)	0.065		
Imipenem	22 (15.7)	16 (3.8)	4.92 (2.52–9.62)	<0.001	0.23 (0.04–1.35)	0.103
Meropenem	16 (11.4)	20 (4.8)	2.56 (1.29–5.09)	0.007	0.70 (0.16–3.13)	0.639
Linezolid	1 (0.7)	2 (0.5)	1.49 (0.13–16.59)	0.744		
Metronidazole	115 (82.1)	319 (76.0)	1.47 (0.90–2.39)	0.121		
Amoxicillin	1 (0.7)	0	/	1.000		
Piperacillin	13 (9.3)	16 (3.8)	2.56 (1.20–5.46)	0.015	0.49 (0.10–2.50)	0.392
Teicoplanin	2 (1.4)	2 (0.5)	3.01 (0.42–21.55)	0.273		
Vancomycin	32 (22.9)	13 (3.1)	9.19 (4.66–18.11)	<0.001	5.69 (1.48–21.78)	0.011
Trimethoprim	1 (0.7)	5 (1.2)	0.59 (0.07–5.12)	0.635		

ASA, American Society of Anesthesiologists score; X±SD-mean ± standard deviation; M-IQR median with interquartile range; H2RA- H2 receptor antagonist; PPI, Proton-pump inhibitors.

### Risk factors for the poor outcome of HAP

The all-cause mortality rate in patients with HAP in this study was 52.1%, significantly higher in the cases than in the group without pneumonia (*p* <0.001). Patients' characteristics, procedures during hospitalization, and characteristics depending on the surgery procedure in the survived and patients who died according to ULRA are shown in Table 3. According to ULRA, the following variables had significant differences between the groups of patients who died compared with those who survived: age, ASA, LOS, MV, and use of ceftriaxone. MLRA identified that age (*p* = 0.040, OR: 0.97, 95% CI: 0.94–0.99), LOS (*p* = 0.006, OR: 1.02, 95% CI: 1.01–1.04), MV (*p* <0.001; OR: 0.20; 95% CI: 0.08–0.47) and the use of ceftriaxone (*p* = 0.046, OR 9.70, 95% CI: 1.04–90.47) were independently associated with poor outcome of HAP.

**Table 3:** Potential risk factors for the outcome of hospital-acquired pneumonia in abdominal surgical patients: results of univariate logistic regression analysis and multivariate logistic regression analysis.

**Variables**	**Survive (*N* = 67)**	**Death (*N* = 73)**	**ULRA crude RR (95%CI)**	**ULRA *p***	**MLRA adjusted OR (95%CI)**	**MLRA *p***
**Patient characteristics**						
Age (X±SD)	61.00 ± 14.62	68.05 ± 14.06	0.97 (0.94–0.99)	0.006	0.97 (0.94–0.99)	0.040
Male (number, %)	39 (58.2)	45 (61.6)	1.15 (0.59-2.27)	0.679		
Diabetes mellitus (number, %)	1 (1.5)	4 (5.5)	0.26 (0.28-2.40)	0.236		
ASA (number, %)			0.27 (0.11–0.67)	0.017	0.39 (0.14–1.11)	0.210
2	21 (31.3)	8 (11.0)				
3	41 (61.2)	58 (79.5)				
4	5 (7.5)	7 (9.6)				
Preoperative infection (number, %)	3 (4.5)	7 (9.6)	2.26 (0.56–9.13)	0.251		
Other postoperative infection (number, %)	25 (37.3)	33 (45.2)	0.72 (0.37–1.42)	0.344		
Malignancy (number, %)	14 (20.9)	10 (13.7)	1.66 (0.68–4.05)	0.262		
**Procedures during hospitalization**						
Length of hospital stay (in days) (M-IQR)	40.00 (29.00–67.00)	35.00 (20.00–50.00)	1.02 (1.00–1.03)	0.020	1.02 (1.01–1.04)	0.006
Hospitalization in ICU (number, %)	56 (83.6)	68 (93.2)	0.37 (0.12–1.14)	0.084		
Length of ICU stay (in days) (M-IQR)	9.00 (1.00–30.00)	17.00 (7.00–28.50)	0.99 (0.97–1.01)	0.194		
Central venous catheter (number, %)	56 (83.6)	64 (87.7)	0.72 (0.28–1.85)	0.491		
Mechanical ventilation (number, %)	32 (47.8)	60 (82.2)	0.20 (0.09–0.43)	<0.001	0.20 (0.08–0.47)	<0.001
H2 receptor antagonist (H2RA) (number, %)	37 (55.2)	38 (52.1)	1.14 (0.58–2.21)	0.707		
Proton-pump inhibitors (PPI) (number, %)	40 (59.7)	34 (46.6)	1.70 (0.87–3.32)	0.121		
Acid suppressive medications (H2RA or PPI) (number, %)	65 (97.0)	66 (90.4)	3.45 (0.69–17.22)	0.132		
Multiple transfusion (number, %)	58 (86.6)	69 (94.5)	0.37 (0.11–1.28)	0.116		
**Characteristics depends of surgery procedure**						
Elective surgery	46 (68.7)	41 (56.2)	0.58 (0.29–1.17)	0.129		
Upper abdominal surgery (number, %)	28 (41.8)	27 (37.0)	1.22 (0.62–2.41)	0.561		
Duration of operation (in minutes) (M-IQR)	120.00 (80.00–170.00)	115.00 (82.50–150.00)	1.00 (1.00–1.01)	0.523		
Class of contamination (number, %)			0.55 (0.27–1.14)	0.108		
Clean/Clean/contaminated	26 (38.8)	19 (26.0)				
Contaminated Dirty/infected	41 (61.2)	54 (74.0)				
Drainage (number, %)	66 (98.5)	69 (94.5)	3.83 (0.42-35.13)	0.236		
Drainage (in days) (M-IQR)	12.00 (8.00–18.00)	12.00 (7.00-18.00)	1.01 (0.98–1.05)	0.404		
Surgical site infection	19 (28.4)	13 (17.8)	1.83 (0.82–4.07)	0.140		
**Antibiotics (HAP therapy)**						
Cefazolin	0	1 (1.4)	0.44 (0.04–5.08)	1.000		
Cefuroxime	0	1 (1.4)	1.54 (0.61–3.88)	1.000		
Ceftriaxone	7 (10.4)	1 (1.4)	8.40 (1.00–70.20)	0.049	9.70 (1.04–90.47)	0.046
Cefepime	2 (3.0)	1 (1.4)	2.21 (0.20–25.01)	0.520		
Amikacin	3 (4.5)	3 (4.1)	1.09 (0.21–5.61)	0.914		
Ciprofloxacin	5 (7.5)	1 (1.4)	5.81 (0.66–51.04)	0.113		
Levofloxacin	2 (3.0)	2 (2.7)	1.09 (0.15–7.98)	0.931		
Ertapenem	5 (7.5)	5 (6.8)	1.10 (0.30–3.97)	0.888		
Imipenem	11 (16.4)	13 (17.8)	0.91 (0.37–2.19)	0.827		
Meropenem	12 (17.9)	16 (21.9)	0.78 (0.34–1.79)	0.554		
Linezolid	1 (1.5)	0	/	1.000		
Metronidazole	21 (31.3)	17 (23.3)	1.50 (0.71–3.18)	0.286		
Amoxicillin	0	0	/	/		
Piperacillin	8 (11.9)	10 (13.7)	0.85 (0.32–2.31)	0.756		
Teicoplanin	2 (3.0)	1 (1.4)	2.21 (0.20–25.01)	0.520		
Vancomycin	15 (22.4)	24 (32.9)	0.59 (0.28–1.25)	0.169		
Trimethoprim	5 (7.5)	1 (1.4)	5.81 (0.66–51.04)	0.113		
Colistin	9 (13.4)	7 (9.6)	1.46 (0.51–4.18)	0.477		
Tigecycline	0	2 (2.7)	/	1.000		

ASA, American Society of Anaesthesiologists score; X±SD-mean ± standard deviation; M-IQR median with interquartile range; H2RA- H2 receptor antagonist; PPI, Proton-pump inhibitors; HAP, hospital-acquired pneumonia.

We registered PN1 (protected specimen bronchial brushing + quantitative culture) in 20 patients (14.3%), PN2 (bronchial aspirate + quantitative culture) in 4 patients (2.9%), PN3 (alternative microbiological criteria - blood culture) in 13 patients (9.3%), PN4 (sputum bacteriology or non-quantitative bronchial aspirate) in 61 patients (43.6%) and PN5 (no microbiological criteria) in 42 patients (37.2%). From the samples of 98 patients with HAP we registered 138 bacterial isolates. In three patients 3 causative microorganisms were isolated, in 34 patients, 2 causative microorganisms were isolated. The most common isolated microorganisms in our patients with HAP were*: Acinetobacter baumannii* (61 patients) followed by *Pseudomonas aeruginosa* (36 patients), *Klebsiella pneumoniae* (15 patients). In addition to the strains listed in the Table 4, the following strains were isolated from the samples of these patients: *Enterococcus faecium* (2), *Proteus mirabilis* (8), *Stenotrophomonas maltophilia* (4), *Staphylococcus aureus* (11) (all isolates of *Staphylococcus aureus* were methicillin-resistant), *Serratia marcescens* (1) and more different isolates that are not considered primary pathogens from samples from which they were isolated (*coagulase negative staphylococci, Corynebacterium* species, *Bacillus* species, *Propionibacterium* species).

**Table 4:** Resistance of isolated Gram-negative bacteria to a selected group of antibiotics in the abdominal surgical patients with HAP in the years 2007–2019.

**Antibiotic Name**	***Acinetobacter baumannii***	***Pseudomonas aeruginosa***	***Klebsiella pneumoniae***
	**(*N =* 61)**	**(*N =* 36)**	**(*N =* 15)**
**Aminoglycosides (** ***n*** **, %)**			
Gentamicin	61 (100)	29 (80.5)	14 (93.3)
Amikacin	61 (100)	25 (69.4)	3 (20.0)
**Carbapenems (** ***n*** **, %)**			
Imipenem	61 (100)	23 (63.9)	0 (0)
Meropenem	61 (100)	21 (58.3)	2 (13.3)
**Cephalosprins (** ***n*** **, %)**			
Ceftazidime	60 (98.4)	22 (61.1)	15 (100)
Cefepime	58 (95.1)	22 (61.1)	15 (100)
Cefotaxime	/	/	15 (100)
Ceftriaxone	/	/	15 (100)
**Fluoroquinolones (** ***n*** **, %)**			
Ciprofloxacin	61 (100)	28 (77.8)	15 (100)
Levofloxacin	57 (93.4)	26 (72.2)	14 (93.3)
**Penicilins (piperacillin) (** ***n*** **, %)**			
Piperacillin	/	20 (55.5)	15 (100)
**Penicilins and** **β-lactamase inhibitors (*****n*****, %)**			
Piperacillin tazobactam	61 (100)	17 (47.2)	14 (93.3)
Ampicillin Sulbactam	37 (60.6)	/	/
**Polymixins (** ***n*** **, %)**			
Colistin	4 (6.6)	/	/
**Other antibiotics (** ***n*** **, %)**			
Trimethoprim	54 (88.5)	/	15 (100)
Tigecycline	/	/	2 (13.3)

n, the number of resistant isolates.

Resistance of isolated Gram-negative bacteria to a selected group of antibiotics are shown in Table 4.

Four or 6.6% of the tested *Acinetobacter baumannii* strains showed resistance to colistin. Also, *in vitro*, 39.4% of strains were susceptible to ampicillin-sulbactam. All *Acinetobacter baumannii* isolates were resistant to aminoglycoside antimicrobial agents and showed carbapenem resistance (CR).

The tested strains of *Pseudomonas aeruginosa* showed the highest sensitivity to piperacillin-tazobactam (52.8%) and the lowest sensitivity to gentamicin (19.5%). More than half of the isolates belong to CR strains (63.9% of the isolates were resistant to imipenem and 58.3% of the isolates were resistant to meropenem). Three isolates showed sensitivity to all tested antimicrobial agents.

The tested strains of *Klebsiella pneumoniae* showed good sensitivity to carbapenem and aminoglycoside antimicrobial agents as well as to tigecycline, while all isolates were resistant to all other groups of antibiotics.

### Antibiotics

Surgical patients with HAP used about 61 DDD per 100 BD more than patients without HAP (107.647 vs. 46.116 DDD/100BD). The most frequently used antibiotics in patients with HAP were vancomycin (23.603 DDD/100BD), followed by metronidazole (18.784 DDD/100BD). Among patients without HAP, the most frequently prescribed antibiotics were metronidazole (14.654 DDD/100BD) followed by ceftriaxone (11.355 DDD/100BD) and cefuroxime 7.908 (DDD/100BD). The consumption of the parenteral antibiotics in patients with and patients without HAP are shown in Table 5.

**Table 5:** The consumption of the parenteral antibiotics in patients with and patients without HAP for the period 2007–2019.

**Antibiotics**	**With HAP**	**Without HAP**
	**DDD per 100 bed days**	
Cefazolin	0.329	1.054
Cefuroxime	4.417	7.908
Ceftriaxone	6.479	11.355
Cefepime	0.732	/
Amikacin	3.582	2.064
Ciprofloxacin	4.626	1.251
Moxifloxacin	/	0.031
Levofloxacin	1.719	/
Ertapenem	3.231	1.283
Imipenem and cilastatin	10.649	1.530
Meropenem	8.293	1.581
Linezolid	0.390	0.072
Metronidazole	18.784	14.654
Rifampicin	0.207	0.103
Amoxicillin	3.051	1.910
Piperacillin	5.476	/
Teicoplanin	0.923	0.164
Vancomycin	23.603	1.086
Trimethoprim	0.446	0.070
Colistin	3.141	/
Fluconazole	5.826	/
Tigecycline	1.114	/
Erythromycin	0.573	/
Total	107.647	46.116

DDD, defined daily dose.

## Discussion

Few studies reported that HAP in patients with abdominal surgical procedures causes significant morbidity and mortality and prolongs hospital stays (14–17). In this study, we analyzed the burden of postoperative HAP in a large cohort of abdominal surgical patients in Serbia. During the study period, 1.6% of all abdominal surgical patients developed HAP in the postoperative period. The frequency of HAP in our patients was higher than reported in the study of Han et al. (27), similar to that reported in the study of Delgado-Rodriguez (28), and lower than in the studies of Mohri (14), Thompson (15), Patel (16) and Evaristo-Méndez (29). These differences could be related to the differences in surveillance methods used, the characteristics of hospital populations studied, and the type of operative procedures conducted.

The CIR of HAP was greatest in the group of patients undergoing small bowel surgery (40.3 per 1,000 operative procedures). This could be explained by the fact that the operative procedures were performed on elderly patients with serious co-morbidities due to emergency conditions caused by mesenteric ischemia and consequent intestinal gangrene, duodenal perforation, etc. Evaristo-Méndez et al. showed that the CIR of HAP was greatest in the group of patients undergoing explorative laparotomy (29), which is the second most common CIR in our patients with of 33.0 per 1,000 surgical procedures. However, one of the studies with the largest number of subjects operated on in the abdominal region reported 16.5 HAP in a sample of 9,054 patients undergoing exploratory laparotomy (15).

Edwards et al. estimated a rate of 6.0 postoperative cases of pneumonia per 1,000 colon surgery procedures (30). Also, a Japanese multicenter retrospective study of elderly patients with colorectal cancer showed that postoperative HAP was registered with the CIR of 24.1 per 1,000 open procedures and 6.0 per 1,000 laparoscopic procedures (18). In our research, open colorectal surgery was the most common operative procedure performed, accounting for 4224 or 43.4 % of the operative procedures with CIR of 9.0 HAP per 1,000 procedures.

Thompson et al. showed that the mean LOS for abdominal surgery patients who developed HAP was significantly greater compared with patients without HAP (*p* <0.001) (15). Also, the prospective multicenter cohort study of major elective abdominal surgery procedures reported that postoperative pulmonary complications, with pulmonary infection as the most common, had the most striking impact on LOS (16). Our study confirmed that LOS was not independent RF for the acquisition of HAP (*p* = 0.533; OR: 1.01; 95%CI: 0.99–1.03), but was independently associated with poor outcome of HAP (*p* = 0.006; OR: 1.02; 95%CI: 1.01–1.04).

HAP is a frequent and severe infection in ICU, with the highest morbidity and mortality (4, 31, 32). Alp et al. (32) reported the rate of HAP and VAP was significantly lower in surgical than in medical ICUs, possibly due to the differences in the proportion of patients who needed MV and the duration of MV. Of our patients with HAP, 88.6% were treated in ICU for more than 48h, and 65.7% needed MV at some time. In the study of ICU-treated patients, Karhu et al. reported that 80% of the HAP patients needed MV (4). Our study showed that ICU and MV were associated with the acquisition of HAP, but didn't retain significance as independent RFs in MLRA. However, the length of stay in the ICU was independently associated with HAP (*p* = 0.002; OR: 1.17; 95% CI: 1.06–1.29) in our surgical patients. Chinese systematic review and meta-analysis showed that stay in ICU is generally longer among Chinese patients with HAP and VAP compared to responding patients in the US (33).

A previous study showed that a restrictive red blood cell transfusion strategy compared with a liberal transfusion strategy was not associated with a reduced risk of HAI infection overall, although it was associated with a reduced risk of serious infection (34). The blood transfusion was independent RFs for the development of postoperative HAP after elective resection of gastric cancer (14). Similarly, in a survey of elective colorectal resections, blood transfusion, surgical wound class, creation of an ostomy, types of operation, ASA score, use of drainage, sex, and surgeon were all important in predicting overall SSI risk (35). Our MLRA identified that the overall risk of HAP was almost four times higher among patients with multiple transfusions (*p* = 0.009; OR: 3.76; 95%CI: 1.39–10.16). The potential benefits and harms of blood transfusion after abdominal surgery should be further studied in clinical prospective studies.

Acid suppressive medication, H2RAs, and PPIs are most commonly used in practice to prevent upper GI bleeding. Herzig et al. (36) found that mentioned drug use was associated with 30% increased odds of HAP. In subset analyses, the risk for HAP was significantly increased with PPIs, but not with H2RAs. On the contrary, a recently conducted Danish systematic review with meta-analysis and trial sequential analysis did not observe evidence for benefit or harm of stress ulcer prophylaxis with PPIs or H2RAs on HAP in adult ICU patients (37). Our patients with HAP received acid-suppressive medications (H2RAs or PPIs) more frequently than patients in the control group (93.6 vs. 79.0%). MLRA identified that overall risk of HAP was significantly higher among patients using H2RA (*p* = 0.007; OR: 4.17; 95%CI: 1.47–11.84), and received acid-suppressive medications (*p* = 0.049; OR: 6.53; 95%CI: 1.01–42.33).

The crude mortality of HAP may be as high as 70% (6). In recent years, studies on attributable mortality of HAI have been relevant, as they quantify the possibility of HAI prevention (38). In their research of attributable mortality, Muscedere et al. (39) focused predominantly on VAP, because of the paucity of evidence on HAP, and concluded that VAP has little effect on hospital mortality but proportionately has a greater impact on LOS and MV, emphasizing that additional research is needed. Recently, the attributable mortality rate of ventilator-associated pneumonia was estimated to be 9% and ranged between 3 and 17% in subgroup analyses (40). In the surgical population, mortality from postoperative HAP ranges from 10.7% (15) to 45% (41). The all-cause mortality rate in patients with HAP in our study was 52.1%, significantly higher in the case than in the control group (*p* <0.001). In our patients, HAP was not the primary cause of death but it was mentioned in the clinical chart information. MLRA identified MV as protective RF for the poor outcome of HAP (OR: 0.20). On the contrary, the U.S. National Surgical Quality Improvement Program database from 2006 to 2012, showed that mortality is significant in patients receiving MV for more than 48 hours who undergo abdominal surgery (42).

The ECDC Point Prevalence Survey conducted in 2011–2012 showed that 18% of HAP cases were defined as laboratory-confirmed based on the case definition code (PN1–PN3) (43). In our study of abdominal surgical patients, 26.5% of patients with HAP were defined as PN1-PN3. Kolpa et al. reported that PN4 was the dominant subtype in ICU patients with a frequency of 79% of all registered HAP (44), while we registered 43.6% of patients with PN4 and 37.2% with PN5 of all HAP among surgical patients.

Studies that analyze the consumption of antibiotics in the treatment of surgical patients with HAP are rare. Meyer et al. showed that halving the duration of treatment for pneumonia in neurosurgical patients results in a reduction of over 30% in antibiotic consumption and costs (45). Also, they calculated that glycopeptide reduction might be associated with a significant decrease in the proportion of methicillin-resistant *Staphylococcus aureus*.

In our abdominal surgical patients with HAP, the most frequently used antibiotic was vancomycin (23.603 DDD/100BD). At the same time, the use of vancomycin in surgical prophylaxis was independent RF for the occurrence of HAP (*p* = 0.011; OR: 5.69; 95%CI: 1.48–21.78). All isolates of *Staphylococcus aureus* were methicillin-resistant, so it would be important to improve the treatment of this group of our patients with possible corrections in the length of glycopeptide administration. Kim et al. showed that inappropriate continued empirical vancomycin use in hospitals with a high prevalence of methicillin-resistant *Staphylococcus aureus* represented 24.9% of the total amount of vancomycin used. Among other things, the empirical use of vancomycin was independently associated with the absence of documented etiological organisms (46). However, Choe et al. (47) reported that interventions such as direct communication with prescribing physicians and infectious disease clinicians can help reduce the inappropriate continued empirical use of vancomycin in Seoul National University Hospital.

Analysis of antibiotic consumption and microbiological epidemiology in surgery departments in Romania hospital in 2017 showed that the most commonly prescribed antimicrobial was ceftriaxone. In this study, *Enterobacterales* (*E. coli, Enterobacter* spp., *Klebsiella* spp., *Serratia* spp., *Citrobacter* spp.), on which ceftriaxone is active, were isolated in 57.92% of cases, but annual sensitivity rates to ceftriaxone were quite low (48.73%) (48). In our patients without HAP secondly most frequently prescribed antibiotic was ceftriaxone (11.355 DDD/100BD) and all tested strains of *Klebsiellae pneumoniae* were resistant to this group of antibiotics.

Liu et al. demonstrated that in patients with CR *Acinetobacter baumanii* bacteremia RF for attributable mortality included stay in ICU, APACHE II scores of >20, respiratory tract as the origin of bacteremia, and use of ceftriaxone prior to the onset of bacteremia (49). Our study showed that the usage of ceftriaxone as therapy was independently associated with poor outcome of HAP (*p* = 0.046, OR 9.70, 95% CI: 1.04–90.47).

Because of all the above, it is important to rationalize the use of antibiotics and administer them according to the microbiologic documentation of infections (6), as well as to implement comprehensive infection control measures, including hospital staff education, contact precautions isolation, environmental cleaning, and hand hygiene promotion in our hospital (50).

The strength of our study is that the data were prospectively collected during 13 years of surveillance of almost ten thousand abdominal surgical patients. Moreover, the strengths of the study include its matched case-control study designs (control patients were matched to the cases by age, gender, ASA score, and type of surgical operation). Also, data could be generalized to patients with abdominal surgical procedures because this study is a “real-life” study. To our knowledge, this is the first study conducted in a cohort of patients undergoing abdominal surgical procedures that comprehensively analyzes risk factors for the occurrence and outcome of HAP, antibiotic consumption, as well as their microbiological causes, and antimicrobial resistance. The present study has several limitations. It is a single-center study. Unfortunately, data concerning patients after discharge from the hospital were not available. Therefore, the frequency and impact of post-discharge HAP and their poor outcome could be underestimated. Also, we did not include some parameters, namely existing of chronic obstructive pulmonary diseases and other chronic lung diseases, alcohol and tobacco use, so analyzing these factors could have enhanced the relevance of our results.

## Conclusion

In conclusion, the incidence of HAP is not low in patients with abdominal operations. It is important to understand its epidemiology and find out opportunities for preventive strategies for HAP and their poor outcome. Our study showed that the independent RFs for the occurrence of HAP are other postoperative infections, the length of ICU stay, the use H2RAs, the use of acid-suppressive medications (H2RAs or PPIs), multiple transfusions as well as the use of vancomycin in surgical prophylaxis. On the other hand, the age, LOS, use of MV and ceftriaxone in HAP therapy, were independently associated with the poor outcome of HAP. The most common isolated microorganisms in our patients with HAP were *Acinetobacter baumannii* and *Pseudomonas aeruginosa*. The most frequently used antibiotics in patients with HAP and without HAP were vancomycin (23.603 DDD/100BD) and metronidazole (14.564 DDD/100BD), respectively. Because of the significant relationship between RFs and HAP and their outcome, the healthcare workers should try to prevent HAP, minimize the use of antibiotics as empiric treatments, as well as to implement comprehensive infection control measures, in abdominal surgery to improve survival.

## Data availability statement

The raw data supporting the conclusions of this article will be made available by the authors, without undue reservation.

## Ethics statement

The studies involving human participants were reviewed and approved by the Ethics Committee of the Military Medical Academy approved the research protocol (MF VMA 14032018). The patients/participants provided their written informed consent to participate in this study.

## Author contributions

Conceptualization, methodology, resources, and project administration: ÐT and VŠ. Software and formal analysis: NR. Validation and writing–review and editing: ÐT, ZK, NR, BR, and VŠ. Investigation: ZK, VŠ, ÐT, and NL. Data curation and supervision: VŠ. Writing—original draft preparation: ÐT, ZK, BR, and VŠ. Visualization: NL. All authors contributed to the article and approved the submitted version.
